# Роль эстриола в лечении атрофии слизистой оболочки нижних отделов мочеполового тракта в постменопаузе

**DOI:** 10.14341/probl13198

**Published:** 2022-12-20

**Authors:** Е. Н. Андреева, Е. В. Шереметьева

**Affiliations:** Национальный медицинский исследовательский центр эндокринологии; Московский медицинский медико-стоматологический университет им. А.И. Евдокимова; Национальный медицинский исследовательский центр эндокринологии

**Keywords:** эстриол, вульвовагинальная атрофия, генитоуринарный синдром, постменопауза, недержание мочи

## Abstract

Исследования последних десятилетий показывают неуклонный рост средней продолжительности жизни человека, и женщины в частности. Всемирная организация здравоохранения прогнозирует к 2030 г. четырехкратное увеличение численности женщин старше 70 лет, при этом многие из них в возрасте после 45 лет могут столкнуться с проблемами климактерического периода. Климактерий является физиологическим состоянием в жизни женщины, в течение которого на фоне возрастных изменений происходят постепенное снижение и выключение функции яичников и прекращение выработки эстрогенов. Генитоуринарный синдром встречается у каждой третьей женщины в этом периоде. Эстриол — основной эстроген, который таргетно решает проблемы, обусловленные эстрогенной недостаточностью: диспареунии, сухости и зуда во влагалище и нижних отделах мочеполового тракта, нарушения мочевыведения, умеренного недержания мочи, а также рецидивирующего вульвовагинита и цистита. Вульвовагинальная дистрофия у женщин старшей возрастной группы — это мультидисциплинарная проблема на стыке гинекологии, урологии и дерматологии, которая может и должна быть решена для предотвращения более тяжелой гинекологической и урологической патологий.

## Abstract (en)

Studies of recent decades show a steady increase in the average life expectancy of a person, and women in particular. The World Health Organization predicts a four-fold increase in the number of women over 70 by 2030, and many of them over the age of 45 may face menopausal problems. Menopause is a physiological state in a woman’s life, during which, against the background of age-related changes, there is a gradual decrease and shutdown of ovarian function and the cessation of estrogen production. Genitourinary syndrome occurs in every third woman in this period. Estriol is the main estrogen that specifically addresses problems associated with estrogen deficiency: dyspareunia, dryness and itching in the vagina and lower genitourinary tract, urinary incontinence, moderate urinary incontinence, and recurrent vulvovaginitis and cystitis. Vulvovaginal dystrophy in women of the older age group is a multidisciplinary problem at the intersection of gynecology, urology and dermatology, which can and should be solved to prevent more severe gynecological and urological pathologies.

Современная демографическая ситуация характеризуется увеличением продолжительности жизни, а следовательно, и ростом популяции пожилых людей. В России количество женщин пери- и постменопаузального возраста составляет более 21 млн [1].

Согласно мировым статистическим данным, средний возраст наступления менопаузы у женщин во всем мире составляет 48,8 года с колебаниями этого показателя в зависимости от географического региона проживания, в РФ он колеблется от 49 до51 года [2–4]. К 2030 г., по демографическим прогнозам, более 1,2 млрд женщин вступят в период менопаузы [2].

Климактерический период — физиологический период, в течение которого на фоне возрастного снижения и последующего «угасания» функции яичников происходит биологическая трансформация различных функций женского организма, изменяется работа отдельных структур центральной нервной системы, активность вегетативной нервной системы, повышается риск развития сердечно-сосудистых осложнений, в значительной мере обусловленных снижением сосудистой эластичности [1].

В настоящее время медицинская общественность проявляет все больший интерес к актуальному междисциплинарному направлению — anti-age медицине (медицине «антистарения»), целями которой являются индивидуализация выявления инволюционных изменений организма и комплексный междисциплинарный подход при их коррекции.

Симптомы генитоуринарной атрофии, согласно мировой статистике, есть у 50% женщин в возрасте 55–80 лет, но только 11% из них будут говорить о ней как о жалобе и проблеме [3], согласно данным по европейской популяции, один и более симптомов вульвовагинальной атрофии (ВВА) имеют до 57,5% пациентов и не получают при этом никакого, в т.ч. патогенетического, лечения [4]. Интенсивность симптомов ВВА имеет прямую корреляцию со снижением качества жизни женщины в мено-и постменопаузе [5].

Не стоит забывать, что гипоэстрогенные состояния на уровне гениталий, в том числе связанные с гипоталамической аменореей или первичной недостаточностью яичников и с явлениями симптоматической ВВА, могут возникнуть у женщин любого возраста [6]. Факторами риска ВВА принято считать: менопаузу, двустороннюю овариэктомию, преждевременную недостаточность яичников, курение, злоупотребление алкоголем, половое воздержание и снижение половой активности, отсутствие родов через естественные родовые пути, другие причины низкого уровня эстрогена (например, послеродовой период, гипоталамическая аменорея), лечение онкологических заболеваний, включая лучевую терапию органов малого таза, химиотерапию и гормональную терапию [7]. Вульвовагинальные симптомы в менопаузе могут быть следующего генеза: инфекционного, воспалительного, психологического (и быть продолжением проблемы в репродуктивном периоде), как правило, это 2 причины и более. Поэтому клиницисту необходим тщательный сбор анамнеза для верификации причины жалоб и разработки индивидуального подхода к лечению конкретной причины.

С какими же жалобами может прийти пациентка на прием к гинекологу в постменопаузе, помимо «приливов»? Как правило, женщина будет предъявлять жалобы на сухость и зуд во влагалище, боль при половых актах, учащенное болезненное мочеиспускание, частые обострения хронического цистита, недержание мочи при позыве. На первый взгляд может показаться, что это проблема урологическая, но все-таки следует помнить: происходит модификация скелетной мускулатуры мышц тазового дна, и следовательно, основной причиной урогенитальных проблем будет несостоятельность мышц тазового дна на фоне эстрогенного дефицита [8][9]. Как мы видим, проблема ВВА при генитоуринарном синдроме у женщин старшей возрастной группы мультидисциплинарная.

Диагноз основывается на базе индивидуального консультирования. Иногда пациентка точно и четко описывает симптомы ВВА, но часто возможно диагностировать эту патологию путем сбора разнообразных жалоб, которые в первую очередь свидетельствуют о снижении качества жизни женщины [10]. В Европе существует опросник вагинального старения (DIVA) — это структурированная шкала для опроса, предназначенная для оценки состояния здоровья влагалища в рамках повседневной жизни женщин, которое отражает эмоциональный комфорт, сексуальную функцию, самооценку и удовлетворение [11]. В РФ такой опросник пока не получил валидации русскоязычной версии.

Идеальная профилактика ВВА у женщин старшей возрастной группы — это поддержание достаточного уровня эстрогенов в пременопаузальном возрасте, этого возможно достичь только в рамках персонифицированного консультирования [3]. В реальной клинической практике, к сожалению, мы это видим крайне редко, и речь идет уже о необходимости не превентивной, а терапевтической медицины. По данным статистики, лечение как в рамках менопаузальной гормональной терапии (МГТ), так и местными эстрогенами начинается крайне поздно (когда клиническая картина уже наиболее яркая) [4]. Нужно отметить, что и сами женщины не считают нужным сказать о жалобах, которые они называют следующим образом: «у всех так», «мне стыдно, я смущаюсь», «старею, не молодею». Генитоуринарный синдром в мено- и постменопаузе остается крайне недооцененной проблемой женщин старшей возрастной группы [12].

Согласно клиническим рекомендациям Международного общества по менопаузе (2020), выбор терапии зависит от тяжести симптомов, эффективности и безопасности лечения для конкретного пациента и его потребностей. Негормональные методы лечения, доступные без рецепта, способствуют облегчению для большинства женщин при легком течении ВВА в менопаузе. Низкие дозы вагинальных эстрогенов, вагинального дегидроэпиандростерона (ДГА)1, системная терапия эстрогенами иоспемифеном1 являются эффективными методами лечения умеренного и тяжелого течения ВВА в менопаузе. При введении низких доз вагинального эстрогена, ДГА или оспемифена прием гестагена не показан. В настоящее время недостаточно данных для подтверждения безопасности вагинального эстрогена, ДГА или оспемифена у женщин с ВВА в менопаузе и раком молочной железы (необходима консультация онколога) [13]. Согласно европейскому протоколу (2020), золотым стандартом лечения ВВА в менопаузе является эстрогенсодержащая терапия, использование которой рекомендуется при умеренных и тяжелых степенях ВВА в менопаузе, которые не купируются негормональными методами. Можно использовать в сочетании снегормональными методами лечения [12].

При этом, согласно протоколу использования локальных форм эстрогенов для лечения постменопаузальной ВВА (EMAS, 2021), самые низкие задокументированные дозы с доказанной эффективностью — это эстриол 30 мкг (0,03 мг) и эстрадиол 4 мкг (0,04 мг) при введении 2 раза в неделю. Абсорбция зависит от дозы, а для продуктов с эстриолом отсутствует преобразование в более мощные эстрогены, такие как эстрадиол или эстрон [44]. Эффективность локальных форм эстриола дозозависима. EMAS clinical guide (2021) ссылается на исследование Griesser H., 2012: двойное слепое плацебо-контролируемое исследование эффективности суппозиториев, содержащих 0,2 мг и 0,03 мг эстриола, где большее улучшение наблюдалось при дозе 0,2 мг эстриола [45]. В исследованиях изучались суточные дозы локальных форм эстриола от 0,5 до 1 мг. Также EMAS (2021) ссылается на систематический обзор C. Rueda, где были показаны эффективность и безопасность эстриола для лечения ВВА у женщин в постменопаузе [46]. Используемые суточные дозы эстриола в метаанализе Cardozo L.D. составили от 0,5 до 3,5 мг. В Кохрейновском обзоре (2016) при ВВА были включены суточные дозы от 0,5 мг до 1 мг [47].

Учитывая разнообразие доз эстриола, используемых в препаратах, нами была разработана классификация доз эстриола, зарегистрированных в РФ, по аналогии с системными формами эстрогенов, представленная в табл. 1.

**Table:** Таблица 1. Классификация доз эстриола, зарегистрированных в РФ Table 1. Classification of daily doses of local estriol *Не зарегистрирован в РФ. Используется при лечении пролапса тазовых органов и недержания мочи.

	Ультранизкая доза	Низкая	Стандартная	Высокая
0,03 мг	0,2 мг	0,5 мг	1–5 мг*
Препарат	Гинофлор	Триожиналь	Овестин	
Состав	Эстриол 0,03 мг+лиофилизат Lactobacillus acidophilus (50 мг)	Эстриол 0,2 мг+прогестерон 2 мг+лиофилизированная культура лактобактерий L. casei rhamnosus Doderleini	Эстриол 0,5 мг	

Согласно российскому протоколу (2020), рекомендуемым методом лечения следует считать при отсутствии противопоказаний локальную гормональную терапию:

Протокол подчеркивает, что симптомы часто возвращаются после прекращения лечения [14].

В 2021 г. был проведен анализ 29 рандомизированных клинических исследований с включением более 8 тыс. пациенток для сравнения нескольких методов лечения (негормональных и гормональных), результатами которого стали следующие выводы:

Следует подчеркнуть, что 2/3 женщин с жалобами на ВВА в менопаузе не довольны и не удовлетворены лечением [4]. Как правило, женщины применяют негормональные непатогенетические методы лечения: различные смазки, средства на основе гиалуроновой кислоты и т.д. Женщины, применяющие МГТ, также попали в эту статистическую группу. Согласно клиническому протоколу 2016 г. (Международное общество по менопаузе (IMS)), применение системной МГТ не предотвращает развитие недержания мочи и не имеет преимуществ перед низкодозированными топическими препаратами эстрогенов при ведении пациенток с урогенитальной атрофией или рецидивирующими инфекциями нижних мочевых путей (уровень доказательности В), лечение следует начинать рано, прежде чем произошли необратимые атрофические изменения, его необходимо продолжать для сохранения полученных преимуществ (уровень доказательности В) [17].

Большинство авторов и все клинические протоколы по ведению пациенток с ВВА в мено- и постменопаузе рекомендуют патогенетическое лечение — применение эстрогенсодержащей терапии таргетно, т.е. интравагинально. Почему эстриол, а не другие эстрогены?

Еще с 1990-х гг. было показано, что эстриол, применяемый вагинально:

ВОЗ призывает по возможности переходить на биоидентичные (молекулярно идентичные эндогенным гормонам, вырабатываемым женским организмом) гормональные субстанции у женщин в менопаузе, стремясь к максимальной эффективности и, главное, — безопасности [19].

Нанотехнологии являются междисциплинарной научной областью, которая в настоящее время претерпевает большое развитие. Нанонизированные и микронизированные формы лекарственных средств позволяют улучшить биодоступность, растворимость и стабильность препаратов. Основной областью применения нанотехнологий в медицине является разработка микро- и наночастиц (МЧ, НЧ), содержащих биоактивные молекулы или контрастные вещества, для различных биомедицинских применений. Одной из тенденций современной фармакологии является не столько получение новых биологических активных молекул или их модификаций, сколько создание новых фармакологических форм: микронизированных, нанонизированных, в виде МЧ, НЧ или «троянских» частиц, предназначенных для различного локального введения. По этим причинам микронизация трудно растворимых препаратов является наиболее подходящим решением. Микронизированные препараты могут входить в состав таблеток, спреев, мазей, кремов. Такие препараты используются для лечения широкого спектра заболеваний (в кардиологии, пульмонологии, гинекологии) [20–22]. Микронизация эстриола позволяет не только решать вопросы на уровне тканей вульвы и влагалища, но и оказывать протективный эффект на нижние отделы мочевой системы. Интравагинальное введение улучшает урогенитальную атрофию и симптомы недержания мочи, что отмечено при кольпоскопиии измерении уретрального давления [25]. Исследование 206 женщин в постменопаузе, которые интравагинально вводили эстриол (начиная с 1 мг в день)3 и применяли реабилитацию тазового дна, показало облегчение симптомов урогенитального старения: улучшение результатов по данным кольпоскопии, достижение максимального уретрального давления, отмечены давление закрытия уретры и коэффициент передачи абдоминального давления на проксимальный отдел уретры [26]. Эстриол (0,5 мг через день3) был столь же эффективен, как эстрадиол1, вводимый через вагинальное кольцо, для облегчения симптомов недержания мочи [27]. Положительный эффект при урологической патологии отмечен в клинических рекомендациях 2020 г.: «Рекомендуется для уменьшения выраженности недержания мочи проводить пациенткам постменопаузального возраста с недержанием мочи вагинальную терапию эстриолом (в виде лекарственных форм для местного применения), при наличии симптомов вульвовагинальной атрофии» [9].

За счет каких же еще механизмов мы видим протективный таргетный эффект эстриола? Матричные металлопротеазы (ММП-2 и -9) и катепсины ответственны за разрушение коллагена у женщин в постменопаузе. Увеличение дисфункции фибробластов — одна из важнейших причин влагалищной атрофии, и терапия местным эстрогеном направлена на восстановление выработки коллагена и подавление экспрессии MMП (к сожалению, не влияет на стрессовое недержание мочи). При сравнении различных схем лечения (ДГА1, предшественниками эстрогена, тестостероном1, местным эстрогеном) было выявлено, что более высокая плотность коллагена наблюдалась именно на фоне терапии местными эстрогенами [33]. Следует помнить, что у женщин с выпадением влагалища происходят замена коллагена I типа на коллаген III типа, редифференцировка гладких мышц в миофибробласты и мышечные архитектурные изменения наблюдались в передней стенке влагалища. Потеря коллагена связана как с гипоэстрогенией, так и с генетическими факторами [34][35].

Референтный лекарственный препарат — лекарственный препарат, который используется для оценки биоэквивалентности или терапевтической эквивалентности, качества, эффективности и безопасности воспроизведенного лекарственного препарата. В качестве референтного лекарственного препарата для медицинского применения обычно используется оригинальный лекарственный препарат. Следует подчеркнуть, что все положительные эффекты, описанные выше, касаются вполной мере оригинального препарата с микронизированным эстриолом. Воспроизведенный лекарственный препарат (дженерик) — лекарственный препарат для медицинского применения, который имеет эквивалентный референтному лекарственному препарату качественный состав и количественный состав действующих веществ в эквивалентной лекарственной форме, биоэквивалентность или терапевтическая эквивалентность которых соответствующему референтному лекарственному препарату подтверждена соответствующими исследованиями. Взаимозаменяемый лекарственный препарат — лекарственный препарат с доказанной терапевтической эквивалентностью или биоэквивалентностью в отношении референтного лекарственного препарата, имеющий эквивалентные ему:

Согласно Информационному письму специалистам здравоохранения от 01.06.2022 г., опираясь на государственный документ (Взаимозаменяемые лекарственные препараты (п. 4 ч. 4 ст. 3 Федерального закона от 27.12.2019 № 475-ФЗ, п. 8 постановления Правительства от 05.09.2020 № 1360)), «в обновленный (дополненный) Перечень взаимозаменяемых лекарственных препаратов вошли нижеперечисленные лекарственные препараты, не являющиеся взаимозаменяемыми референтному лекарственному препарату, эстриол, крем вагинальный, 1 мг/сут (Овестин): крем вагинальный Орниона, вагинальные суппозитории Овипол Клио, вагинальные суппозитории Эстрокад» [31]. Эти важные аспекты важно и нужно учитывать лечащему врачу для достижения максимальной эффективности в лечении и, конечно же, безопасности проводимой терапии.

Таким образом, механизм положительного влияния эстриола при интравагинальном использовании заключается в следующем:

«Девизом» клинических рекомендаций NICE (2015) стала фраза «интравагинальный эстроген можно использовать столько, сколько нужно» [30].

Российский клинический протокол и международные клинические рекомендации подчеркивают, что если речь идет только о наличии ВВА у женщины в мено- и ранней постменопаузе, то системная МГТ не у всех и не всегда решит проблему, поэтому предпочтение отдается интравагинально вводимому эстриолу [4–5][13][29][31].

Все больше появляется исследований по положительному влиянию андрогенов при ВВА у женщин в мено- и постменопаузе. Данные об андрогенном воздействии на эпителиальный слой противоречивы в рамках ВВА (т.к. рецепторы к андрогенам расположены на проксимальных концах нервных окончаний, присутствуют в эпителиальном слое и собственной пластинке, а также в мышечном слое). Недостаток тестостерона считается причиной уменьшения смазки влагалища и сексуальной дисфункции, однако изначально местное применение тестостерона привело к уменьшению кератинизации и увеличению муцификации. Необходимы дополнительные исследования, чтобы определить безопасность, дозозависимость данной терапии [35–38].

Также активно обсуждается применение интравагинального геля с ДГА1 при ВВА, который связан с локальной ароматизацией андростендиона и тестостерона в эстрон (E1) и эстрадиол (E2). После менопаузы секреция эстрогена яичниками прекращается, а уровень его в сыворотке остается в биологически неактивных концентрациях, в результате чего ДГА становится единственным источником эстрогенов и андрогенов, превращенных в периферических тканях [35][38–41]. В Российской Федерации ДГА зарегистрирован только в виде БАД.

Интравагинально вводимый эстриол при ВВА в мено- и постменопаузе соответствует основным четырем основополагающим принципам медицины: персонализации (врач и пациент выбирают удобную форму для каждого: крем или суппозитории), предикции (врач проговаривает с пациенткой факторы риска ВВА для усиления эффекта от терапии), превентивности (своевременное назначение местной терапии эстрогенами в необходимой дозе), партисипативности (индивидуальное консультирование и донесение мысли о длительности терапии — вовлекает пациентку в процесс терапии). Данные принципы также отражены в российских клинических рекомендациях:

## ЗАКЛЮЧЕНИЕ

Более трети своей жизни женщина проводит в состоянии дефицита женских половых гормонов. По данным ВОЗ, в большинстве стран мира продолжительность жизни женщин после 50 лет колеблется от 27 до 32 лет. С каждым годом возрастает число женщин, вступающих в период менопаузы. Менопауза, не являясь собственно заболеванием, приводит к нарушению эндокринного равновесия в организме женщины, вызывая «классические» проблемы: вазомоторную симптоматику, нарушения психологического здоровья, урогенитальные расстройства, остеопороз, сердечно–сосудистые заболевания. Эстриол, ввиду его специфических особенностей действия на таргетные органы и отсутствия пролиферативного влияния на эндометрий имолочные железы, является одним из эффективных, приемлемых и безопасных методов терапии при различных гинекологических состояниях, связанных с эстрогенным дефицитом слизистой оболочки нижних отделов мочеполового тракта в постменопаузе. Рандомизированные клинические исследования показали не только эффективность локальной эстрогенотерапии в лечении ВВА как в режиме монотерапии, так и в комбинации с системной МГТ, но, главное, — безопасность данного вида терапии.

## ДОПОЛНИТЕЛЬНАЯ ИНФОРМАЦИЯ

Источники финансирования. Работа выполнена по инициативе авторов без привлечения финансирования.

Конфликт интересов. Авторы декларируют отсутствие явных и потенциальных конфликтов интересов, связанных с содержанием настоящей статьи.

Участие авторов. Все авторы одобрили финальную версию статьи перед публикацией, выразили согласие нести ответственность за все аспекты работы, подразумевающую надлежащее изучение и решение вопросов, связанных с точностью или добросовестностью любой части работы.

1. Не зарегистрирован в РФ. 2. Оспемифен (торговые марки Osphena и Senshio производства Shionogi) является селективным модулятором рецепторов эстрогена (SERM), действующим аналогично эстрогену на эпителий влагалища, увеличивая толщину стенки влагалища, что, в свою очередь, уменьшает боль, связанную с диспареунией. Препарат был одобрен FDA в феврале 2013 г. и Европейской комиссией по маркетингу в ЕС в январе 2015 г. Не зарегистрирован в РФ. 3. Режим дозирования, не зарегистрированный в РФ.
